# Assessing Acceptability of Biodegradable Contraceptive Implants in Kenya and Senegal

**DOI:** 10.9745/GHSP-D-23-00503

**Published:** 2024-08-27

**Authors:** Alice F. Cartwright, Rebecca L. Callahan, Anna Lawton, Christina Wong, Oliver Muchiri, Samira Matan

**Affiliations:** a Product Development and Introduction, FHI 360, Durham, NC, USA.; b noodle research, Durham, NC, USA.; c Global Health and Population, FHI 360, Durham, NC, USA.; d ThinkPlace, Nairobi, Kenya.; e ThinkPlace, Dakar, Senegal.

## Abstract

**Background:**

Contraceptive implants are popular in Africa, but barriers to removal exist. Biodegradable implants (BDIs) offer an alternative to the need for removal. This study explored potential user, provider, and other stakeholder perspectives on 2 BDI prototypes, revealing opportunities and challenges for introduction.

**Methods:**

We conducted focus group discussions (FGDs) and in-depth interviews (IDIs) with women, men, family planning (FP) providers, community influencers, and FP policymakers and program staff in Kenya and Senegal. Characteristics of the 2 BDI prototypes were shared, and participants held and interacted with placebo prototypes. Structural coding was used to analyze the data focused on key product attributes, including biodegradation, removal potential, size, material, insertion site, and duration of effectiveness.

**Results:**

We conducted 16 FGDs and 35 IDIs with 106 participants in Kenya and 15 FGDs and 43 IDIs with 102 participants in Senegal. Overall, respondents liked the idea of a BDI, noting the avoidance of pain and scarring and reduced transport and costs as benefits of no removal requirement. Kenyan respondents expressed greater understanding of the biodegradation process than those in Senegal, though potential users in both countries expressed concerns about possible side effects associated with the process. In Senegal, mention of cholesterol in a BDI caused concern, while Kenyan participants responded positively to the same BDI being composed of organic materials. The second BDI product was viewed as more similar to existing implants, which providers preferred. Participants suggested increasing the pregnancy protection duration beyond 18 months. No clear preference between products emerged, and participants liked and disliked some characteristics of both.

**Conclusions:**

Kenyan and Senegalese participants expressed interest in the BDI concept but expressed some reservations related to biodegradation, material, and side effects. BDIs offer the opportunity to expand contraceptive choice. However, messaging around product characteristics will be required for successful introduction and uptake.

## Abstract (precis)

Biodegradable contraceptive implants under development offer the potential for expanded choice for long-acting contraception with the benefit of no removal. Introduction and marketing efforts will need to consider messaging around product characteristics.

## Teaser

**Key Findings:**

- Contraceptive implants are a popular method in Kenya and Senegal, but access to removal continues to be a challenge that could be partially alleviated by biodegradable implants (BDIs).
- Potential users, health care providers, and other stakeholders like that BDIs would not need to be removed, which would save time and money for users and health systems, as well as reduce pain and scarring from removal.
- Concerns about biodegradation and possible side effects were raised by participants in both Kenya and Senegal.

**Key Implications:**

- User feedback on BDI prototypes can help refine product characteristics to be more acceptable before the method is brought to market.
- As BDI products are further refined, additional information will be needed from low- and middle-income countries to determine the system-level needs and user-focused positioning for successful introduction.

## BACKGROUND

Contraceptive implants are a key part of the global contraceptive method mix and have increased in popularity around the world over the past decade.^1^ An estimated 23 million women worldwide currently use implants as their contraceptive method, a population that has increased tenfold since the mid-1990s.^2^ Implant use in sub-Saharan Africa, in particular, has grown significantly since 2013, largely as a result of significant negotiated price reductions for Jadelle and Implanon/Nexplanon through volume guarantee agreements for Family Planning 2020 (FP2020) focus countries.^3^^,^^4^

However, as implant use has increased, gaps in access to quality and timely implant removal services have surfaced, especially in low and middle-income country (LMIC) settings. Barriers identified include a lack of trained providers at facilities, insufficient supplies, provider refusal to remove, user fees, lack of information communicated to the user regarding the length of effective use of the implant or where to go to obtain removal services, distance to the facility, and access to affordable transportation.^5^^–^^10^

Unlike current implants, biodegradable contraceptive products currently in development would not require removal. Such products would ideally be removable for a period of time after insertion but would ultimately degrade in the user’s body at the end of the period of effectiveness. The introduction and uptake of such a product could reduce the number of people seeking implant removals.

The introduction and uptake of a biodegradable contraceptive product could reduce the number of people seeking implant removals, which remain a challenge to access in LMICs.

The idea for such a product is not new. Clinical trials for 2 contraceptive biodegradable implant (BDI) products were conducted in the United States in the 1980s and 1990s, but neither progressed to market.^11^^,^^12^ These early studies focused on the clinical performance of the prototypes and collected little data on potential user acceptability of a BDI and none from LMIC settings.

In the last decade, donors and product developers have shown renewed interest in the BDI concept. FHI 360 currently has 2 BDIs in its pipeline at different stages of development. One is a cholesterol and etonogestrel (ENG) fused pellet formulation known as Casea S developed in collaboration with pH Sciences and Gesea Biosciences, and the other is a rod-shaped implant consisting of a novel biodegradable polymer core developed by researchers at Yale University surrounded by a shell of levonorgestrel (LNG). The currently available removable implants on the market also contain ENG (Nexplanon) or LNG (Jadelle and Levoplant). Both BDI product leads are designed to prevent pregnancy for 18 months and be removable for the first 12 months. In 2016, preliminary research in Uganda and Burkina Faso explored reactions to the general BDI concept and revealed interest in the product but also concerns about its degradation in the body and potential side effects.^13^ In this study, we aimed to explore in more depth potential user, provider, and other stakeholder perspectives on the 2 BDI prototypes currently under development and to assess opportunities and challenges for eventual product introduction.

## METHODS

### Study Setting and Approach

We conducted this research in Nairobi and Meru counties in Kenya and Dakar and Thiès regions in Senegal. These 2 countries were chosen to capture East and West African country experiences, and the specific counties/regions selected included urban, peri-urban, and rural populations. In addition, Kenya and Senegal have different overall contraceptive use rates, but implants make up a relatively high proportion of the method mix.^14^^,^^15^

To understand how these new BDI products might be perceived and accepted by potential users and other stakeholders, we conducted a descriptive and rapid qualitative market research project guided by human-centered design (HCD) principles. HCD is an approach to problem-solving that develops solutions by involving the human perspective in all steps of the process. Human involvement typically takes place by initially observing the problem within context (empathizing), brainstorming (ideating), developing concepts (prototyping), assessing the relevance and resonance of concepts with intended users (testing), and finally implementing a tested solution. Its emphasis on user participation, projective techniques/activities, and implicit information gathered through immersive fieldwork attempts to generate a more comprehensive understanding of challenges and codesign solutions alongside stakeholders to increase acceptability and sustainability.

We conducted in-depth interviews (IDIs), focus group discussions (FGDs), and included HCD activities intended to capture a diversity of perspectives in different settings. IDIs are widely known to be excellent at collecting detailed individual perspectives with little/less social influence, while FGDs are good at achieving collaborative conversations and potentially revealing social norms in a more concentrated way. We used both to go both deep and broad in our FGDs as well as achieve perspectives from more participants than IDIs could achieve alone. We also favored smaller FGDs to encourage trust and candor, given the sensitive nature of the conversations involving sex/sexuality and contraception.

### Participant Eligibility and Recruitment

Study participants in both countries were recruited to represent 5 distinct populations: (1) potential future BDI users; (2) men whose partners were currently using a modern method of contraception besides sterilization; (3) health care providers trained in contraceptive implant insertion and removal; (4) community leaders/influencers; and (5) family planning (FP) policymakers and program staff (referred to as key informants). We chose to interview participants from these groups because contraceptive acceptability, preferences, and use are influenced by many people, including those who provide information but also reinforce social norms. For the potential users, we used purposive sampling to select participants who were either current or previous implant users or those who had never used implants but were willing to consider using hormonal contraception. In addition, for each of the 2 potential BDI user groups, we sought representation of younger (18–24 years) and older (25–49 years) age groups. Potential users were eligible if they were not sterilized and were not trying to get pregnant. Key informants were identified based on their involvement in decision-making regarding FP guidelines, implementation, and programs in the country. This included government officials focused on FP at the national and regional levels and people working on FP for nongovernmental organizations at the national level.

Eligibility requirements for all participant groups included age 18 years or older (18–49 years for potential users), willingness to be photographed and audio recorded (as part of the documentation process), and ability to give informed consent to participate. Local mobilizers, including community health volunteers, identified potential users, men, health care providers, and community leaders in all study areas and, using a recruitment script, invited them to participate. Key informants were contacted by staff from ThinkPlace (a design and strategy consulting firm) in collaboration with FHI 360 staff in both countries.

### Data Collection Activities

Trained staff from ThinkPlace collected data in July 2022 in Kenya and January 2023 in Senegal. Subsequently, due to challenges with recruiting sufficient participants from all target groups, specifically younger FP users and Ministry of Health officials, additional Senegalese data collection was conducted in May 2023. IDIs and FGDs were carried out in a mix of languages by country and region according to participant preferences: Kiswahili and English (Nairobi), Kiswahili and Kimeru (Meru), and Wolof and French (Thiès and Dakar). Staff followed semistructured interview guides tailored for each participant group. First, for all groups except key informants, the IDIs and FGDs incorporated interactive activities, including dissolving bouillon cubes or vitamin C/glucose tablets, for participants to uncover local terms and context-specific understanding of what the degradation process entailed. Then, all participant groups were presented with the 2 BDI product concepts, referred to as Casea S and the Yale rod (Figure), and allowed to handle placebo prototypes. Topics discussed by participant group are outlined in Table 1.

**TABLE 1.** Topics Covered During In-Depth Interviews and Focus Group Discussions on BDIs, by Participant Group, Kenya and Senegal

**Participant Group**	**Topics Covered**
Potential users (Current/previous implant users and never implant users)	Understanding of degradation as a concept Characteristics of BDIs: Size Proposed insertion location Visibility/palpability Duration Possible side effects Removability Any suggested changes
Men	Understanding of degradation as a concept Community beliefs What types of women they would advise to use BDIs Any suggested changes
Community influencers	Understanding of degradation as a concept Community beliefs What types of women they would advise to use BDIs Any suggested changes Best communication channels for future method introduction
FP providers	Experience with current implants Understanding of degradation as a concept Characteristics of BDIs: Size Proposed insertion location Visibility/palpability Duration Possible side effects Removability How they would counsel on BDIs Any concerns about removal process Any suggested changes
Key informants (FP policymakers and FP program staff)	Acceptability among users Feasibility of introduction in the national health system and/or facilities Procurement and stocking concerns

Abbreviations: BDI, biodegradable implant; FP, family planning.

**FIGURE:**
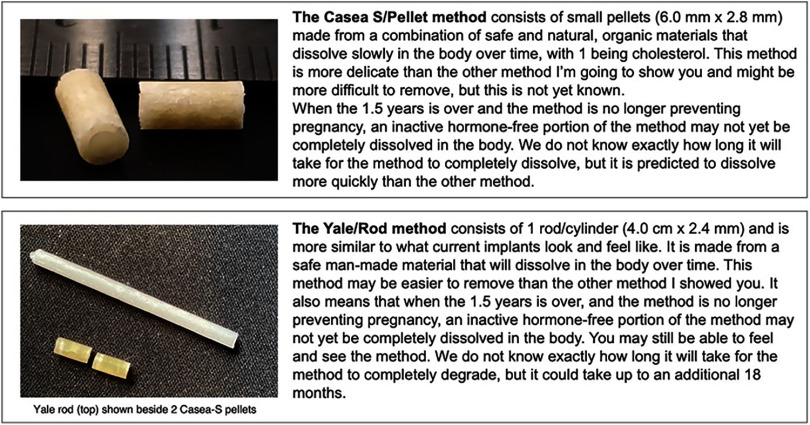
Descriptions of Biodegradable Implants Presented to Research Participants

All IDIs and FGDs were conducted in private rooms of health facilities in each county/region (except for the key informant interviews) and were audio-recorded, along with notetaking by the research team. Key informant interviews were conducted in the offices of the informants, except for 2 key informants in Senegal who were not able to meet in person and returned their responses to the questions in the discussion guide by email. All participants gave their written consent, and all potential participants (except for key informants) received compensation for the cost of their transportation to the health facilities.

### Data Analysis

We used detailed notes from both countries, as well as transcriptions of the audio recordings in Senegal, to populate data extraction tables in Excel with the same topic areas as the discussion guides. The tables were completed in English in Kenya and French in Senegal. Subsequently, 2 analysts (AC and CW) drafted memos for Kenya based on topic themes for which the data had been entered into the data extraction table. Because the data extraction table was not completed in as much detail for Senegal compared to Kenya, a third analyst (AL) did a more in-depth coding of all source documents from Senegal and then drafted memos to mirror the Kenyan documents. For both countries, all data were reviewed to determine patterns and trends, and findings were described with textual summaries, attribution tables, and illustrative quotes, where possible. Because many of the documents were notes providing summaries of the IDIs and FGDs rather than transcripts, verbatim quotes were not available in every instance.

### Ethical Approval

The study received ethical approval from the National Commission for Science, Technology, and Innovation and the Amref Ethics and Scientific Review Committee in Kenya, the Comité National d’Ethique pour la Recherche en Santé and the Commission de Protection des Données Personnelles in Senegal, and FHI 360’s Protection of Human Subjects Committee in the United States.

## RESULTS

We conducted 16 FGDs and 35 IDIs with a total of 106 participants in Kenya and 15 FGDs and 43 IDIs with a total of 102 participants in Senegal (Table 2).

**TABLE 2.** Participants in Research Activities in Kenya and Senegal

		**Kenya**				**Senegal**			
		**Nairobi, No.**		**Meru, No.**		**Dakar, No.**		**Thiès, No.**	
		**Activity**	**Participants**	**Activity**	**Participants**	**Activity**	**Participants**	**Activity**	**Participants**
Current/previous implant users	FGD	6	29	3	17	8	33	1	5
	IDI	5	5	8	8	6^a^	6	9^a^	9
Never implant users	FGD	--	--	1	4	1	3	--	--
	IDI	4	4	1	1	4^a^	5	5^a^	6
Men	FGD	1	6	1	4	1	2	--	--
	IDI	4	4	4	4	3^a^	3	1	1
Community influencers	FGD	2	4	--	--	--	--	1	3
	IDI	1	1	4	4	1	1	3	3
FP providers	FGD	1	5	--	--	1	3	2	8
	IDI	2	2	1	1	2	2	2	2
Key informants	FGD	1	2	--	--	--	--	--	--
	IDI	1	1	--	--	7^a^^,^^b^	7	--	--
Total	FGD	11	46	5	25	11	41	4	16
	IDI	17	17	18	18	23	24	20	21
			63		43		65		37

Abbreviations: FGD, focus group discussion; FP, family planning; IDI, in-depth interview.

^a^ Second round of data collection included IDIs with 2 current/previous implant users, 5 never implant users, 1 man, and 3 key informants in Dakar and 1 current/previous implant user and 2 never implant users in Thiès. Two of the additional IDIs with never implant users had 2 participants each.

^b^ Two key informant IDIs in Senegal emailed their responses to the discussion guide.

### Degradation as a Concept

Participants in Kenya were more able than those in Senegal to describe what was happening when shown the example of a bouillon cube or vitamin/glucose tablet dissolving in water. They alternately described the process as dissolving, disappearing, or melting, using local terms, including kuyeyuka (to dissolve or disappear in Kiswahili) and gukeruka (to melt in Kimeru). Current/previous implant users in Kenya most often referenced dissolvable sutures as a similar process, though this was also mentioned by providers and 1 male partner in Senegal.

*When I gave birth, I did it through the cesarean section method and the doctors used stitches after that. These stitches disappeared and I did not have to go to hospital to have them removed as they just disappeared in the body. —Participant in FGD of current/previous implant users, Nairobi, Kenya*

More current/previous implant users and 1 never implant user in Senegal compared the process to tablets or painkillers, which were more often mentioned by providers and 1 community influencer in Kenya. Some respondents noted that they did not worry when they took these medicines because even if they were not sure where it went, they knew it worked.

*As with effervescent tablets, when it dissolves in a glass of water the medicine remains, and when you drink it, it heals what it needs to heal in the body - it disperses and enters the blood [stream].* —Current injectable user, Thiès, Senegal

Other less frequently cited comparative processes included injections (more commonly mentioned in Senegal), sweets, tea and sugar, and washing powder.

*When a woman is cleaning clothes, she puts Omo [brand name of washing powder] in water and the Omo disappears, yet the clothes get clean. The BDI is the same; it will dissolve in the body and prevent a woman from getting pregnant.* —Community influencer, Nairobi, Kenya

However, several participants in both countries raised a concern about the idea that the BDI would be degrading in the body.

*It’s not like pills which you swallow, where is the implant dissolving to?* — Participant in an FGD of current/previous implant users, Meru, Kenya

Another Kenyan current/previous implant user wondered if the BDI would lose its effectiveness as it dissolved. Participants in both countries also raised some benefits and concerns associated with the biodegradation process, described in more detail in the sections on removability and side effects.

### Feedback on Biodegradable Implant Product Characteristics

Participants provided feedback on the characteristics of the 2 BDIs overall, as well as specific feedback on the individual Casea S and Yale products based on the descriptions provided and their handling of the inert prototypes.

#### Size/Number

In both countries, more potential users expressed a preference for the size and number of rods of the Yale BDI. Reasons included that it looked more like implants currently available, only required 1 incision that would presumably limit pain and scarring (compared to the perception that Casea S would require multiple incisions for the pellets, though this was not how the Casea method was presented), and fears that Casea S was too small and may disappear in the body. More providers in both countries also thought the Yale BDI would be easier to insert (and remove if necessary) and easier for users to palpate to confirm placement. In addition, a provider in Senegal noted that Casea S’s different appearance compared to current implants might affect the uptake among potential users.

*The size is a problem, it [Casea S] is too small. [It would be better] if it were the same size as the implants that clients are used to seeing. With this size, it risks [raising] a lot of questions and we will have to have valid arguments to convince the users, which will also require a lot of work from service providers.* —Participant in FGD with FP providers, Dakar, Senegal

Though fewer, some potential users (more in Senegal) and providers saw some benefits of Casea S’s smaller size, especially as it related to discretion.

*The Casea S may even be confused for a pimple on the skin due to its small size; hence no one will pay much attention to it being on the woman’s arm.* —FP provider, Nairobi, Kenya

Some users also mentioned that Casea S’s small pellets might be easier and less painful to insert, with smaller incisions and less scarring.

#### Material

Participants in Kenya noted that the material of the BDIs seemed harder than current implants, which they feared might make them more visible once inserted (mentioned in 1 FGD of current/previous implant users) or painful when a user moves their arm (mentioned in 1 provider FGD). Some respondents were also concerned that these “harder” implants would be more brittle and could break once implanted in a user’s arm. However, the fact that the Casea S was described as made from “organic” materials particularly resonated with Kenyan community influencers and providers.

*The organic material that constitutes the Casea S is highly preferred owing to the fact that government has been encouraging use of herbal/organic products…People believe that natural products have minor side effects compared to plastics or man-made materials.* —FP provider, Nairobi, Kenya

In some of the Senegal interviews, the descriptions of the 2 BDIs were presented incorrectly by the data collection team, with 1 described as being made of synthetic material with cholesterol and the other made of organic material. This conflation of the materials and possible lack of clarity that the amount of cholesterol present in Casea S is minimal made it difficult to draw conclusions about participant preferences for the 2 different materials. Nevertheless, the majority of comments about the BDI materials from Senegalese potential users, providers, and other respondents mentioned concerns about the presence of cholesterol specifically. Providers mentioned that this would be a contraindication for some potential users.

*Cholesterol also poses a bit of a problem here in Senegal, with our way of eating, and especially in hypertensive people.* —FP provider, Dakar, Senegal

However, a few Senegalese potential users mentioned that the concept of organic materials was perceived to be reassuring and safer.

In both countries, a few providers mentioned concerns about potential interactions of the BDI materials with other substances. In Senegal, a provider wondered if the hormones in the BDI would interact with the cholesterol and affect the mechanism of action, while a Kenyan provider questioned whether the anesthesia used during implant insertion or removal could cause the BDI to be impacted and reduce its effectiveness.

#### Visibility/Palpability

Almost all potential users in both countries expressed a desire for the BDIs to be palpable but not visible. The desire for palpability was to ensure correct and continued placement and that the BDI had not migrated to another part of the body.

Almost all potential users in both countries expressed a desire for the BDIs to be palpable but not visible.

*I feel [for the method] all the time to reassure myself that the implant is in place because I have a friend who has lost the implant in her arm. She’s gained weight and [now it’s] lost in her arm. So far she hasn’t had any children and the doctors can’t find it.* —Participant in FGD of current implant users, Thiès, Senegal

Visibility, on the other hand, was not desired, as it could be perceived as indicative of being very sexually active or having an affair. Providers echoed these concerns in both countries, saying that women would want to be able to palpate the BDIs to reduce fears of migration, and it helped providers to know if implants were placed correctly after insertion.

*Those who hide [that they use] FP will prefer it because it can’t be seen. [But] psychologically it can also cause a problem for those women who wish to feel it all the time. Sincerely, it will be very difficult.* —Participant in FGD of FP providers, Thiès, Senegal

A few potential users in both countries expressed their understanding that the BDI would be less palpable over time, saying that because it was biodegradable, it made sense that at some point, it would dissolve and they would not be able to feel it. One potential user in Senegal said it would be “perfect” not to be able to see it or feel it as long as it was effective at preventing pregnancy.

One concern mentioned in Kenya was a concern about the continued palpability of the nonhormonal portion of the BDIs that might remain in the body and continue dissolving past the 18-month duration of pregnancy protection. Potential users, community influencers, and key informants mentioned that the continued palpability may be confusing and give people incorrect reassurance that they are still protected against pregnancy.

*For current implants, people assume that since they can feel the implant, that the implant is still effective. It will be important to sensitize users that having the nonhormonal portion after the 18 months does not mean that the implant is effective.* —Current implant user, Meru, Kenya

#### Insertion Location

Most potential users in both countries thought the upper arm would be a preferred insertion location, as it is where current implants are inserted and therefore familiar, and that location would not bother users or be visible to others. The close second most mentioned location was the thigh, as potential users noted that it would be a discreet location that they could still monitor themselves.

*Why not change to an insertion site that will be more discreet, such as in the thigh, for example. Nobody will know if you have an implant or not.* —Current implant user, Dakar, Senegal

Other locations mentioned in Kenya included the buttocks (because people are used to receiving injections there) and the wrist.

#### Duration

Most potential users in Kenya were amenable to an 18-month duration of pregnancy prevention for the BDIs. The duration was perceived as not too short that the user could forget to go back for another method but also not too long to have to deal with side effects if they occurred. All Kenyan providers also agreed that the 18-month duration was a good middle ground for those who did not want to use current implants for their full duration.

*Currently, for users who want to have a 2-year method, we usually advise them to have the 3- or 5-year implant inserted and then come for removal after the 2 years. With a 1-and-a-half-year method, then we will be able to address clients like these who do not prefer short or long durations.* —FP provider, Nairobi, Kenya

While a few potential users in Kenya said they would prefer a shorter duration to try the BDI and see if they liked it, more said they would prefer a longer duration. Most of these respondents were characterized by wanting no more children and not wanting to keep going back to the health facility. Similarly, 1 community influencer and most men and other respondents in Kenya felt that 18 months was too short. Some FP policymakers and program implementers thought that the duration should be changed to 2 years to align with World Health Organization (WHO) recommendations on birth spacing.

Potential users, providers, community influencers, and other respondents in Senegal also suggested that a longer duration, such as 2 or 3 to 5 years, would be preferred.

*The [duration] is too short. 18 months is nothing. A normal pregnancy spacing is 2 years, but for me, [I want a] minimum of 3 years. Our mothers and grandmothers [adhered to] 2 years of pregnancy spacing, but times have changed. For me, it is necessary to space at least 3 to 5 years to be able to properly educate your child and put them in good conditions before getting pregnant again*. —Participant in FGD of current implant users, Dakar, Senegal

However, many Senegalese providers and a few community influencers also said that 18 months was “good enough,” as it was close to the WHO-recommended 2 years for spacing. Only 1 potential user in Senegal said the duration should be shorter.

#### Removability

Many potential users and community influencers in both countries liked the idea that women could avoid the pain, scars, and costs that came with implant removal, as well as save time. However, many potential users also noted they had concerns about the possible high cost of seeking care at a higher-level facility if they needed removal, as well as possible pain and scars that might come with a more complicated removal of a partially degraded implant.

Many potential users and community influencers in both countries liked the idea that women could avoid the pain, scars, and costs that came with implant removal, as well as save time.

*For me the nonsurgical removal part makes me feel okay because sometimes we can’t afford to pay for removal procedures so this makes it efficient, but I also fear that it will take a higher person [health provider] in case I have to remove it—that also gives me a bit of fear.* —Never FP user, Nairobi, Kenya

Potential users in Senegal raised concerns about possible side effects of the implant remaining and dissolving in the body without being able to remove it and said they would want it to be removable for the full period of effectiveness. However, a few others noted that a year to remove was sufficient as it would allow ample time to experience side effects and remove if needed. Reasons mentioned for desiring removability for the full period included removal at the request of a partner or to prove their fertility in a new relationship. In contrast, in Kenya, a respondent liked that the BDI would have a period of protection for which it could not be removed.

*Since I cannot have the BDI removed after 12 months, it would be very easy to say “no” to my husband in case he ever insists that I get another child say after a year after insertion, as there really is nothing I can do at that point in time.* —Participant in FGD of current/previous implant users, Nairobi, Kenya

Most providers in both countries felt that a year time frame for removal was sufficient because if users were going to experience side effects, they would likely occur in the first year. A few Senegalese providers and community influencers noted that the 12-month removal window was fine, but it would impact the counseling they would provide on the method. One community influencer provided an example of how she would counsel potential BDI clients.

*If you want to remove [the method] you have to come and do it during the first year but if it exceeds the 1 year, then the process of degradation begins. Patience is required for the 6 months that remain while the dose ends in your body.* —Community influencer, Thiès, Senegal

All providers in Kenya were fine with removal requiring a higher-level facility, and a few even noted that it would reduce their workload. However, a couple of providers mentioned they would need additional training to conduct removals. Some Kenyan providers echoed the benefits that potential users could avoid pain and costs associated with implant removal. A few providers, a community influencer, and a key informant in Senegal also voiced concerns raised by potential users in that country—namely that the inability to remove after some time could be problematic for people being pressured by external sources like their partners. In addition, they reiterated that people might fear the impact on their fertility if they were unable to remove them.

*The fact that it dissolves in the body, women will ask if they can still get pregnant. Being able to remove reassures women. They will panic if told they cannot remove [the method] after 1 year.* —Participant in FGD of community influencers, Dakar, Senegal

#### Side Effects

In both countries, potential users raised concerns about side effects, particularly related to how the degradation of the BDI would impact the body. They mentioned a possible accumulation of chemicals in the blood from the dissolving BDI.

*So I’m already thinking of the simple fact we’re not going to remove it. I’m ready for that but am also thinking of the consequences of degradation. Of knowing that it’s going to stay in your body. Isn’t that going to have any consequences in your system? So that’s what it’s really about. That’s my concern. I tell myself that it can mix in the blood and create problems.* —Current implant user, Dakar, Senegal

Potential users, community influencers, and key informants also spoke about possible impacts on menstruation and return to fertility (as noted in the section on removability). Concerns about amenorrhea were mentioned as they relate to beliefs about blood building up in the body, though 1 Kenyan key informant noted that some users prefer amenorrhea because they save money on menstrual products. More providers in Kenya said that return to fertility would not be an issue as long as it was communicated clearly to potential users, whereas potential effects on fertility were more of a concern among Senegalese providers and community influencers. A Senegalese key informant compared the experiences of DMPA users to how the BDI product might impact return to fertility.

*After 1 and a half years, there can be an incomplete dissolution of the product - a residue of the product still in the body - which delays the return to fertility. That we already [have with] DMPA… We normally tell you that the product is for 1 and a half years and at the end … she can get pregnant. But after the year and a half she can’t get pregnant, she mustn’t believe that she’s been sterilized.* —FP program implementer, Dakar, Senegal

A few Kenyan potential users also mentioned concerns that the nonhormonal portion of the BDI could have negative side effects if it remained in the body and was still dissolving (even after the effective portion had already dissolved).

Overall, most potential users in both countries said that they would want more information about the possible side effects they might experience with the BDI before deciding to use it, with a smaller number noting that they would be reassured if the product was well-researched and side effects were known. In addition, a few potential users echoed providers in thinking that any side effects would likely be experienced in the first 12 months, so removability for that period was satisfactory. A few potential users who had never used implants in Senegal and providers in both countries questioned whether there would be unique side effects or interactions due to the materials or the degradation of the BDIs. However, most Senegalese providers said that if the BDIs had similar side effects to existing implants, they knew how to counsel on and treat them.

### Ideal Target Clients and Considerations for Specific Populations

Most community influencers and men in Kenya said that anyone could use a BDI. Those participants who made a distinction about who should use BDIs (in both countries) specifically mentioned its duration of effectiveness, saying that BDIs might appeal most to those who think 3–5-year methods are too long. Some of these groups might include newlyweds, those who prefer short-acting methods, those who had miscarriages, and wives of migrant workers or in long-distance relationships. In addition, respondents thought the shorter length would be good for younger women who want more children but want to space their pregnancies.

*[The duration] is a big change in relation to existing methods. I tell myself it can be perceived positively because women who had reluctance that a method will last 5 years [might be interested].* —FP program implementer, Dakar, Senegal

Other benefits of BDIs for specific populations were highlighted by providers in Kenya. Adolescents and people experiencing gender-based violence would not have to return to the health facility for method supply (as needed for short-acting FP methods) or removal (as is the case for current implants) and would not have to get money for removal. Not having to return to the facility for removal was also noted as a possible benefit for people with disabilities who might need assistance for mobility. A Kenyan provider underscored that they would give the same counseling on the BDI to all clients, including on return to fertility, as they would not know about a person’s marginalized identity unless they were outwardly living with disabilities.

### Preferences for Biodegradable Implant Characteristics and Suggested Changes

#### Overall Likes and Dislikes

Overall, respondents in both countries liked that the BDIs did not have to be removed, thus saving women from the pain and scars associated with removal and time in returning to the facility for removal (Table 3). Providers also felt that the products could reduce their overall workload and help in situations where not all providers were trained to remove implants. Though mentioned less frequently, the perception that Casea S was made of more “natural” materials was appealing to Kenyan participants. While respondents in both countries liked the smaller, potentially more discreet Casea S, they also liked that the Yale BDI looked more similar to existing implants, giving potential users, community influencers, and providers the impression that it would be easier to insert and remove if needed. More respondents in Kenya thought that the 18-month proposed duration was a good middle ground between current short-acting and long-acting methods.

**TABLE 3.** Preferences for Biodegradable Implant Products Currently in Development

	**Likes**	**Dislikes**
Casea S	“Natural” materials (Kenya only) Smaller size (more discreet, may dissolve faster, less painful to insert)	Contains cholesterol (Senegal only) More difficult to palpate due to small size More difficult to insert and remove multiple pellets
Yale	Looks more similar to existing implants (1 rod) Easier to insert and remove if needed Potentially easier to palpate	Larger size (more painful to insert) Perceived brittle nature of material based on prototype “Harder” material could be easier to palpate and/or see by others
Both products	No incision or scars for removal 18 months is not too long or too short (more common in Kenya)	High costs for removal Side effects, especially those that might be caused by degradation Non-hormonal portion remaining in body longer (Kenya only) 18 months not long enough (more common in Senegal) Unable to be removed for entire duration (more common in Senegal)

Providers felt that the BDIs could reduce their overall workload and help in situations where not all providers were trained to remove implants.

While natural materials appealed to Kenyan participants, those in Senegal focused on the cholesterol in Casea S as a point of concern. In addition, respondents in both countries feared the small size of Casea S could make it more difficult for providers to palpate to ensure placement after insertion and for users to monitor for migration. A perception was also voiced that it might be more difficult to insert and remove the multiple Casea S pellets. The main concern regarding the Yale BDI was that it might be more painful to insert due to its (relatively) larger size. Other concerns raised about both products were possible higher removal costs due to their potential need to be removed at a higher-level facility if partially degraded and side effects, especially those that might be unique or made worse by the degradation process. Kenyan respondents were more likely to bring up concerns about the nonhormonal portion of the implant remaining in the body beyond 18 months, while respondents in Senegal were more likely to feel that an 18-month duration was too short and that removal should be possible for the full duration.

Based on the positive and negative characteristics attributed to the 2 BDI products, respondents made suggestions for changes to the BDI products (Table 4).

**TABLE 4.** Suggested Changes to Biodegradable Implant Products

**Characteristic**	**Suggested Change**
Size	Combine Casea S into 1 pellet Make Yale rod smaller/shorter
Material	Make softer/more flexible Make both products out of “natural” materials (Kenya only) Remove/reduce cholesterol (Senegal only) Ensure non-hormonal portion also dissolves in 18 months or short time after (Kenya only)
Visibility/ palpability	Palpable throughout duration (Senegal only)
Duration	Increase duration to 2–3 years or 3–5 years (more common in Senegal, less in Kenya) Reduce duration to 6 months to 1 year, or provide different options for duration (fewer respondents mentioned)
Removability	Removability for full duration (more common in Senegal, less in Kenya)
Side effects	Reduce side effects, especially menstrual changes

Some final points made by respondents about the future introduction of BDI products in their countries were related to education and sensitization. Particularly in Kenya, potential users, community influencers, and providers underscored the need to ensure public sensitization with multiple audiences, especially conducted by health care providers and community health volunteers. Kenyan providers also suggested that wall charts to describe and differentiate the 2 BDI products would be useful, as well as possible video demonstrations of BDI users sharing their experiences with use, side effects, and removal that could be used during counseling.

## DISCUSSION

This qualitative market research study revealed that potential users and providers in Kenya and Senegal are interested in the concept of BDIs but have concerns about the product prototype characteristics, particularly related to biodegradation, implant material, palpability, removability, and side effects. Many of the concerns raised were associated with both BDIs, and we did not find an overwhelmingly clear preference for 1 product over the other. Study participants’ feedback on method characteristics points to potential changes that could make the BDIs more appealing, as well as how they might eventually be introduced and marketed in different settings.

One key concern mentioned that differed across the 2 settings related to the material comprising Casea S. The Casea S implant was described as being made of “a combination of safe and natural, organic materials that dissolve slowly in the body over time, with 1 being cholesterol.” This description was received well in Kenya, and the concept of something being “organic” particularly resonated with participants. In contrast, in Senegal, participants focused on the mention of “cholesterol” and expressed concern about how it might impact potential users who have high cholesterol. While it is highly unlikely that the cholesterol in Casea S would increase cholesterol levels in a user, this description of the product raised concerns. Existing contraceptive implants are not generally contraindicated for users with hypertension (or high cholesterol).^16^ However, providers should always discuss existing health issues with potential users to help determine appropriate methods. Recognizing that the mention of cholesterol may raise user concerns is also important when considering how to describe the BDI materials in future marketing/positioning efforts and what specific terminology resonates in different contexts.

Participants in both countries were interested in the concept of BDIs but had concerns about biodegradation, implant material, palpability, removability, and side effects.

Participant responses to issues of BDI size and removability are likely informed by experiences with existing implants in both countries. For example, even though some liked the small size of Casea S for discretion, many potential users in both countries were reassured that the Yale product looked more like existing implants, and providers felt that it would be easier to insert and remove since it was 1 long rod. Familiarity with existing implants may also be reflected in the preferences that Senegalese participants expressed about removability, with many potential users saying that they wanted a product that could be removed for the full duration. Additional research may be needed in Senegal, comparing the time frame for which a BDI cannot be removed to the experience of injectable contraception, for example, to further explore acceptability and differentiate the BDI from existing implants.

The main BDI characteristic that appealed to participants in both countries was that they do not have to be removed, avoiding pain and scarring, as well as transport and procedure costs. This response was similar to reactions to the concept of BDIs for HIV pre-exposure prophylaxis expressed by young people in South Africa, who had strong preferences for fully dissolvable biodegradable implants to reduce clinic visits and avoid painful removals.^17^ Providers in the present study, as well as the pre-exposure prophylaxis BDI study, expressed that patients not having to return to the facility could reduce their workload as well as potentially save health care costs.^17^ However, in both studies, potential users expressed concerns about possible side effects from something dissolving in the body. Even though Kenyan participants in the present study expressed more understanding of the concept of degradation than their Senegalese counterparts and provided more real-world examples of the process, they still expressed reservations about possible side effects associated with degradation. Earlier research in Burkina Faso and Uganda also documented concerns about where the contents of the implant go after dissolution and if they could have negative effects on fertility.^13^ Future clinical studies of BDIs will need to confirm whether side effects are similar to those of existing progestin-only contraceptives.

Providers expressed that patients not having to return to the facility could reduce their workload and potentially save health care costs.

Navigating the competing issues of biodegradation and palpability could present a challenge in the further development of BDIs, especially as it relates to a nonhormonal element remaining in the body after the effective portion has dissolved. While Senegalese users expressed a preference for palpability of the BDI throughout its use, Kenyan participants were concerned that the continued palpability of the nonhormonal portion may be confusing and lead people to falsely believe that they are still protected against pregnancy. This is in addition to concerns mentioned in prior BDI research where providers worried that a product that “disappears” could reinforce misperceptions that contraceptive implants migrate in the body.^13^ Clear communication on the effectiveness period will be essential, similar to counseling provided with existing implants: that they are no longer effective after 3 or 5 years, even though they can still be palpated.

Finally, this study revealed variation in duration preference for the BDI products, with more support for the proposed 18-month period of effectiveness in Kenya than in Senegal. Respondents in Senegal were more likely to want a longer duration, though participants in both countries wondered if a 2-year duration might be better to align with WHO birth spacing recommendations.^18^ These differing opinions echoed those from previous research, where an 18-month duration was mentioned as both a potential advantage to use for spacing births and disadvantage because the duration was not long enough.^13^^,^^19^ However, the intermediate duration is still a unique feature of BDI products and one that was mentioned as particularly useful for younger women who still want more children. This may be a population of particular interest for future marketing and communication materials for BDI products.

The results of this research align with what we know to be true about every contraceptive—1 size does not fit all, and no method will appeal to all users. The variety of preferences for BDI characteristics, especially length of pregnancy prevention, may present a challenge for pharmacological development and design. For example, for a longer duration of pregnancy prevention, more active drug ingredients may be needed that may result in a larger size of the BDI, which would then make that characteristic less attractive to users. However, BDIs will offer removal-free long-acting contraception, a prospect that will undoubtedly be attractive to some users and many providers. As the products move through clinical development and closer to market, focused effort will be required to describe and position these products for maximum uptake. Future directions for research could include discrete choice experiments, which allow for the comparison of specific product characteristic options and could help further refine based on potential user preferences.

### Limitations

This study had some limitations that are worth mentioning. First, the study was not intended to be representative of the opinions of all potential users, providers, and other stakeholders in either country, and it is possible that other respondents in other areas of each country would have provided different opinions and experiences. We also experienced some delays and challenges with data collection in Senegal, which resulted in fewer participants recruited than originally planned in that setting and incorrect description of the BDI material in some interviews. In line with HCD principles, ThinkPlace also conducted 1 codesign workshop in each country with a subset of initial FGD and IDI participants. However, detailed notes from the Senegal workshop were not available, and therefore, any additional insights from those workshops are not captured in the results presented here. Finally, due to participants handling the BDI prototypes in the FGDs and IDIs, there was some documented degradation of the physical prototypes. It is possible that the appearance of the prototypes may have influenced participants in later FGDs and IDIs to remark on the fragility of the BDI products.

## CONCLUSION

While contraceptive BDIs are still several years away from entering the market, the results of this research may be useful for current global health practice. Biodegradable products are being developed in other therapeutic areas, such as HIV prevention. The lessons from this research may inform introduction strategies for such products. Additionally, those working in FP service delivery may be interested to know that new biodegradable contraceptives are on the horizon. Market introduction of a novel FP method like a BDI will take considerable planning and coordination for adequate financing, procurement planning, market positioning, and provider training, among other factors. The results of this research may help FP programs and funders to begin thinking through the requirements needed for successful introduction.

## Notes

**Peer Reviewed**

**First Published Online:** July 16, 2024.
